# Human Cytomegalovirus Primary Infection and Reactivation: Insights From Virion-Carried Molecules

**DOI:** 10.3389/fmicb.2020.01511

**Published:** 2020-07-14

**Authors:** Yu-Qing Wang, Xiang-Yu Zhao

**Affiliations:** ^1^Peking University People’s Hospital, Peking University Institute of Hematology, National Clinical Research Center for Hematologic Disease, Key Laboratory of Hematopoietic Stem Cell Transplantation, Beijing, China; ^2^PKU-THU Center for Life Sciences, Academy for Advanced Interdisciplinary Studies, Peking University, Beijing, China

**Keywords:** HCMV, virion-carried molecules, primary infection, reactivation, tegument, envelope

## Abstract

Human cytomegalovirus (HCMV), a ubiquitous beta-herpesvirus, is able to establish lifelong latency after initial infection. Periodical reactivation occurs after immunosuppression, remaining a major cause of death in immunocompromised patients. HCMV has to reach a structural and functional balance with the host at its earliest entry. Virion-carried mediators are considered to play pivotal roles in viral adaptation into a new cellular environment upon entry. Additionally, one clear difference between primary infection and reactivation is the idea that virion-packaged factors are already formed such that those molecules can be used swiftly by the virus. In contrast, virion-carried mediators have to be transcribed and translated; thus, they are not readily available during reactivation. Hence, understanding virion-carried molecules helps to elucidate HCMV reactivation. In this article, the impact of virion-packaged molecules on viral structure, biological behavior, and viral life cycle will be reviewed.

## Introduction

Human cytomegalovirus (HCMV), officially referred to as human herpesvirus 5 (HHV5), is one of nearly 100 known herpesviruses and is subclassified as a beta-herpesvirus. The seroprevalences vary with respect to socioeconomic background. Generally, the virus is widespread (Cannon et al., 2010), and serum positive for infection in the general population has been estimated to be 83% (Zuhair et al., 2019). Moreover, seronegativity does not show a complete correlation with negativity for HCMV DNA in CD34^+^ hematopoietic progenitor cells (HPCs) (Khaiboullina et al., 2004). Transmission of primary infection usually occurs via intrauterine (Boppana and Britt, 1995), breast milk (Hayes et al., 1972), and contaminations exposure routes (such as saliva or genital secretions) (Khaiboullina et al., 2004; Murata et al., 2009). Reactivation occurs from latency, the sources of which are CD34^+^ HPCs and CD14^+^ monocytes (Mendelson et al., 1996), often after blood transfusion and organ transplantation (Zhao et al., 2017). HCMV infection in healthy individuals is often mild or asymptomatic. By contrast, it is highly pathogenic among congenitally infected infants and immunocompromised patients, such as transplant recipients (Liu et al., 2015). Universal infection of HCMV and persistent lifelong infection suggest that HCMV primary infection has to negotiate a balance with the host. The virion-carried molecules are served as readily available mediators that facilitate viral survival. In this review, the significance of the factors packaged in the HCMV virion will be discussed.

## HCMV Structure

### Genome

The HCMV genome, the largest among nine human herpesviruses, is a linear double-stranded DNA containing 230 to 240 k base pairs (Davison et al., 2003; Dolan et al., 2004; Bradley et al., 2009). The viral genome comprises two unique regions termed unique long (UL) and unique short (US), and these two domains are flanked by a pair of inversely repeated sequences, forming a genome configuration known as TRL-UL-IRL-IRS-US-TRS.

A few hurdles arise when analyzing the HCMV genome sequence and associated virology. First, the laboratory passaged strains AD169 and Towne following subsequent culture accumulate substantial deletions, especially in the UL/b’ region, and compensated insertions of several repeats in the long terminal region in comparison to clinically isolated viruses such as Toledo (Cha et al., 1996). The UL/b’ gene products pUL133–pUL138 are associated with latency, reactivation, and activation of the expression of certain genes in an interdependent manner with pUL97 (Revello and Gerna, 2010; Li et al., 2014). Additionally, the UL/b’ region has been reported to be involved in NK cell evasion (Tomasec et al., 2005). Merlin and Toledo showed stronger ability against NK cell immunity than AD169 and Towne (Tomasec et al., 2005). *In vitro* analysis demonstrated that HCMV strains that harbor UL/b’ were more sensitive to growth defects mediated by maribavir, a pharmacological pUL97 inhibitor (Wang et al., 2013). Together, high-passage strains represent a different biological manner than a wild-type virus. Moreover, the laboratory adaptation behavior differs in different HCMV strains. For example, approximately 13 and 15-kbp sequences in the UL/b’ region were deleted in Towne and AD169, respectively, compared with Toledo (Cha et al., 1996). Second, although the HCMV genome can be cloned and maintained in bacterial artificial chromosomes (BACs), mutations can occur before BAC cloning (Stanton et al., 2010). The Merlin BAC revealed that this low passage strain contains *RL13* and *UL128* mutations, and repair of these mutations led to impaired replication in fibroblasts (Stanton et al., 2010). The *RL13* (Stanton et al., 2010) and *UL128* (Ryckman et al., 2008) genes encode virion glycoproteins. Some patients present a switch in the HCMV population during the course of disease, and the dominantly altered genotypes encompass genes encoding immunomodulatory mediators and glycoproteins (Hage et al., 2017). Those genes may be associated with the pathogenesis of HCMV-caused disease (Hage et al., 2017). *In vitro* studies using viruses harboring mutations in such genes may provide misleading results regarding the natural behavior of clinically important HCMV. Third, without culture, contamination of cellular DNA that is difficult to eliminate from sequencing causes low viral sequence reads in next generation sequencing. Finally, regardless of immune status, the majority of patients and carriers are infected with mixed HCMV strains (Novak et al., 2008; Ross et al., 2011; Jiang et al., 2017). It has been reported that mixed infection with more than one HCMV strain is associated with severe clinical outcomes such as an increased viral load (Manuel et al., 2009) and progression to CMV disease (Coaquette et al., 2004). Although some degrees of genome instability have appeared in HCMV *in vitro*, a genome reference is still needed. The Merlin strain is a low passage strain and is characterized by relative genomic integrity, allowing to more accurately reflect the wild-type clinical isolates, despite some minor changes, for example, the single-nucleotide substitution in the *RL13* and *UL128* gene (Akter et al., 2003; Dolan et al., 2004; Sijmons et al., 2014; Wilkinson et al., 2015).

### Capsid (Table 1)

The viral capsid provides a layer enclosing the genome. It is an icosahedral structure with a triangulation number T of 16, containing 60 asymmetric units (Chen et al., 1999). Sixteen major capsid proteins (MCPs), 16 smallest capsid proteins (SCPs), 5 triplexes (Ta, Tb, Tc, Td, and Te), and 1/3 Tfs exist in each unit. Five or six MCPs form a penton or hexon, with the latter subdivided into P (peripentonal), C (center), and E (edge) hexons as a result of different positions. Near the upper domain of MCP is the SCP. Triplexes are heterotrimers that are composed of Tri1, Tri2A, and Tri2B (Yu et al., 2017). The HCMV genome size is approximately 50% longer than the herpes simplex virus type 1 (HSV-1) genome. The diameter of HSV-1 and HCMV capsid is approximately 125 and 130 nm, respectively (Butcher et al., 1998). The capsid volume of HCMV is slightly larger (about 17%) than HSV-1 (Bhella et al., 2000). The significant increase in HCMV genome size does not correlate with the slightly larger capsid volume (Bhella et al., 2000). Hence, the HCMV genome is tightly packaged within the capsid shell and protected by the capsid. This pressurized packaging of the genome is a reasonable strategy for the nucleocapsid to deliver the viral genome into the nucleus by leveraging the internal pressure within the capsid (Yu et al., 2017; Brandariz-Nuñez et al., 2019).

**TABLE 1:** HCMV capsid proteins.

Protein	Mapped gene	Kinetics	Function	References
Major capsid protein	*UL86*	Late	Component of capsid	Chen et al., 1999; Casavant et al., 2006; Yu et al., 2017
Smallest capsid protein	*UL48/UL49*	Late	Component of capsid	Gibson et al., 1996b
Tri1 (minor capsid binding protein)	*UL46?*	Late	Component of triplex on capsid	Gibson et al., 1996a
Tri2 (minor capsid protein)	*UL85*	Late	Component of triplex on capsid	Gibson et al., 1996a

### Tegument (Table 2)

Tegument is a link layer between the nucleocapsid and outer envelope, largely composed of diverse proteins that appear unlikely to form a definitive structure. The amorphous layer may contain some RNAs (Bresnahan et al., 2000; Greijer et al., 2000; Terhune et al., 2004). Although we were intrigued about the biological significance of RNA package in-between the envelope and capsid, the mechanisms underlying how the RNA is enclosed and how they interact with other elements remains largely unexamined. The tegument components are directly delivered into host cytoplasm, allowing the newly infected virus to adapt to the new cellular environment. In contrast, tegument proteins are not readily utilized by reactivated virus, indicating a difference in primary infection and reactivation. Thus, some tegument proteins that play roles in initial infection are true late proteins, which are not expressed at the early phase of the reactivated virus life cycle and are not supposed to assume responsibilities in primary infection. The diverse effects of tegument proteins on HCMV biology (Table 2) will dominate the following discussions.

**TABLE 2:** HCMV tegument proteins.

Protein	Mapped gene	Kinetics	Function(s)	References
pUL23	*UL23*	Early late	➢ Reduces the STAT-mediated INFγ responses	Adair et al., 2002; Feng et al., 2018
pUL24	*UL24*	Early late and true late	➢ Interacts with other viral proteins	Adair et al., 2002; To et al., 2011
pUL25	*UL25*	True late	➢ Co-localizes with ppUL99 in the perinuclear regions within cytoplasm ➢ Prevents Pul26 from degradation ➢ Interacts with viral proteins and behaves like a helper—a hub.	Battista et al., 1999; Zimmermann et al., 2018
pUL26	*UL26*	Early late	➢ Be a transcriptional activator of major immediate early enhancer–promoter ➢ Participate in phosphorylation and stabilization of pp28 ➢ Limits ISGylation of interferon-stimulated gene 15 (ISG15) ➢ Blocks NF-κB	Stamminger et al., 2002; Lorz et al., 2006; Munger et al., 2006; Kim Y. J. et al., 2016; Zimmermann et al., 2018
pp150	*UL32*	Late	➢ Highly immunogenic and pp150 antigen can be detected by ELISA ➢ Interact with capsomeres and triplex on capsid and directs translocation of nucleocapsid for further envelopment ➢ Be involved in nuclear targeting, organization of assembly compartment, tegumentation and virion egress during late infection ➢ Restricts viral IE gene expression	AuCoin et al., 2006; Indran et al., 2010; Tandon and Mocarski, 2011; Bogdanow et al., 2013; Xi et al., 2017; Yu et al., 2017
ppUL35	*UL35*	Predominantly late	➢ Facilitates a proper recycling, transportation and localization of gB ➢ Cooperates with pp71 to activate viral gene expression	Liu and Biegalke, 2002; Salsman et al., 2011; Maschkowitz et al., 2018
pUL36	*UL36*	Immediate-early	➢ Inhibits caspase-8 activation and thus apoptosis	Skaletskaya et al., 2001; Yao et al., 2004
pUL38	*UL38*	Early and late	➢ Modulates cellular metabolism ➢ Prevents premature cell death by protecting lysosome integrity, relieving endoplasmic reticulum (ER)-mediated apoptosis ➢ Accumulates translational factors by regulating poly(A) binding protein (PABA)	Terhune et al., 2007; Xuan et al., 2009; Qian et al., 2011; McKinney et al., 2012; Sun et al., 2018; Rodríguez-Sánchez et al., 2019
pUL43	*UL43*	Late	➢ Localizes in perinuclear region in cytoplasm	Adair et al., 2002
HCMV DNA polymerase processivity factor	*UL44*	Early and late (sumoylated pUL44 was detected at late phase)	➢ Tethers the catalytic subunit pUL54 onto DNA ➢ Communicates a series of proteins such as cellular nucleolin and viral pUL114 to ensue replication ➢ Acts concomitantly with IE86 to block p53 activity and thus overcomes cell cycle arrest and apoptosis ➢ Binds to interferon regulatory factor 3 (IRF3), disassociates IRF3 with NK-κB and prevents subsequent activation of anti-viral genes	Weiland et al., 1994; Loregian et al., 2003; Strang et al., 2010a; Kwon et al., 2012; Sinigalia et al., 2012; Fu et al., 2019
Inactive homolog of the large subunit of ribonucleotide reductase	*UL45*	Early	➢ Catalyzes the dNTP synthesis ➢ Inhibits RIP1-mediated NF-κB activation	Patrone et al., 2003; Kwon et al., 2017
pUL47	*UL47*	Late	➢ Regulates tegument assembly ➢ Binds to pUL48 binding protein, which is required to deubiquitylating activity of pIL48	Hyun et al., 1999; Tullman et al., 2014; Cappadona et al., 2015
pUL48	*UL48*	Late	➢ Deubiquitinating activity ➢ Cooperates with pUL47 to promote disassembly of nucleocapsid and enhance the release of viral DNA from capsid ➢ Guides pUL47 translocation from nucleus to cytoplasm	Bechtel and Shenk, 2002; Cappadona et al., 2015; Kim Y. E. et al., 2016; Kwon et al., 2017
pUL50	*UL50*	Late	➢ Form the nuclear egress complex ➢ pUL50 may have a role in maintaining HCMV genome during latency	Dal Monte et al., 2002; Rossetto et al., 2013; Lye et al., 2015
pUL53	*UL53*			
DNA polymerase catalytic enzyme	*UL54*	Early	➢ Have polymerase activity, 3′ to 5′ exonuclease activity as well as ribonuclease H activity ➢ Target of antiviral drugs (e.g., ganciclovir)	Zarrouk et al., 2017
Single-stranded DNA binding protein	*UL57*	Early	➢ Cooperates with other replication initiation proteins to promote DNA synthesis	Anders, 1990
pUL69	*UL69*	Immediate-early	➢ Transactivate certain genes, mediate mRNA nuclear export ➢ Binds to UAP56 and URH49, and enhances the nucleocytoplasmic shutting activity ➢ Induces G1 block of host cell cycle	Winkler et al., 1994; Hayashi et al., 2000; Zielke et al., 2011; Tunnicliffe et al., 2018
pUL71	*UL71*	Early	➢ Contributes to secondary envelopment	Schauflinger et al., 2011; Dietz et al., 2018
pUL72	*UL72*	Late	➢ Be a homolog of dUTPase (inactive?)	Caposio et al., 2004; Gopal et al., 2018
pUL76	*UL76*	Immediate-early, late	➢ Regulates *UL77* gene expression ➢ Modulates gene expression (repress viral replication) ➢ Activates DNA damage response	Wang et al., 2004; Isomura et al., 2010; Costa et al., 2013
pUL77	*UL77*	Early late	➢ Binds to dsDNA and terminase subunits	Meissner et al., 2011; Köppen-Rung et al., 2016
pUL79	*UL79*	Early late	➢ Promotes the accumulation of late viral transcripts	Perng et al., 2011a
pp71	*UL82*	Immediate-early	➢ Degrades Daxx ➢ Transactivation MIE gene ➢ Degrades Rb at the onset of lytic infection, promotes cell progression and inhibits apoptosis ➢ Binds to STING and impedes subsequent activation of TBK1 and IRF3, a method against innate immunity ➢ Circumvent surface MHC-I expression	Kalejta and Shenk, 2003; Saffert and Kalejta, 2006; Trgovcich et al., 2006; Torres and Tang, 2014; Fu et al., 2017
pp65	*UL83*	Early late	➢ Binds to cGAS and IFI16 and interferes with innate immunity ➢ Mitigate IL-1β via disrupting NF-κB signaling pathway ➢ Highly immunogenic and elicits adaptive immunity ➢ May regulates cell cycle ➢ Used in the pp65 antigenemia test	Chevillotte et al., 2009; Cui et al., 2009; Arcangeletti et al., 2011; Tandon and Mocarski, 2011; Li et al., 2013; Biolatti et al., 2018a, b
pUL84	*UL84*	Early protein synthesized at late time (Spector and Tevethia, 1994)	➢ Associates with pUL44 and nucleoli in the replication compartments ➢ Involved in initiation of viral replication ➢ Be a transdominant inhibitor of IE2-p86 during gene expression ➢ Facilitates localization of *IRS1* mRNA	Gebert et al., 1997; Colletti et al., 2005; Gao et al., 2010; Bender et al., 2014
pUL93	*UL93*	Early and late	➢ Required for DNA cleavage packaging	Wing and Huang, 1995; DeRussy and Tandon, 2015; Borst et al., 2016
pUL94	*UL94*	True late	➢ Acts as a nucleocytoplasmic shuttling protein and allows correct localization of pUL99 in vAC	Phillips and Bresnahan, 2012; Phillips et al., 2012
pUL96	*UL96*	Early	Assists pp150 to co-stabilize nucleocapsids	Tandon and Mocarski, 2011
pUL97	*UL97*	Early late	➢ Kinase activity (such as phosphorylating (i) antiviral prodrugs, (ii) pUL69 to contributes to the viral mRNA export), (iii) Rb proteins to regulate cell cycle, (iv) nuclear lamina to promote egress ➢ Organizes assembly site ➢ Activate gene expression	Michel et al., 1996; Azzeh et al., 2006; Feichtinger et al., 2011; Li et al., 2014; Sharma et al., 2014; Iwahori and Kalejta, 2017
pp28	*UL99*	Late	➢ Involved in envelope assembly	Silva et al., 2003; Chevillotte et al., 2009
pUL103	*UL103*	Late	➢ Enhances development of cVAC	Lyons et al., 1994; Das et al., 2014
pIRS1/pTRS1	*IRS1/TRS1*	Early late	➢ Inactivates RNA-dependent protein kinase R (PKR) ➢ Interacts with pUL44 ➢ Binds to autophagic proteins and inhibits the antophage ➢ Promotes expression of the replication genes	Iskenderian et al., 1996; Marshall et al., 2009; Strang et al., 2010b; Ziehr et al., 2016
pUS22	*US22*	Early late	➢ Immunogenic and antigenic, and can exert humoral responses	Dal Monte et al., 1998; Adair et al., 2002
pUS24	*US24*	Immediate-early	➢ Activate viral gene expression	Feng et al., 2006

#### DNA Replication

Human cytomegalovirus initiates lytic infection by expression of genes in a flow cascade; immediate-early (IE) phase prior to early gene expression and finally late genes are expressed to facilitate virion assemble and release (Weekes et al., 2014). DNA amplification occurs after early gene expression and before late gene expression. *In vitro* DNA replication usually initiates at 24 to 72 h post infection. *Ori*Lyt, adjacent to *UL57*, occupies a gene region of 2 kb where HCMV DNA replication commences, and there is a region that can regulate both *UL57* transcription and *ori*Lyt activation (Kiehl et al., 2003). Nuclear domain 10 (ND10) is the location for the deposition of viral DNA (Ishov and Maul, 1996), which will be discussed later in the section *Roles of pp71 in primary infection and reactivation*. Replication-associated proteins are recruited to the site around the DNA replication compartment that is derived from budding of peripheral ND10 (Ahn et al., 1999). Six proteins of the replication machinery conserved in members of herpesviridae include pUL54 (DNA polymerase), pUL44 (DNA polymerase processivity factor), pUL57 (single-stranded DNA binding protein), pUL70 (primase), pUL102 (primase-associated factor), and pUL105 (helicase).

An association network is constructed in which pUL44 communicates with a series of proteins to orchestrate viral DNA replication such as cellular nucleolin, viral pUL114, and TRS1 (Strang et al., 2010a, b). UL112-113 gene products p43 and p84 may play a role in the recruitment of pUL44 (Schommartz et al., 2017). HCMV *UL79*, *UL87*, and *UL95* are expressed early and recruited into the pre-replication complex with pUL44 (Isomura et al., 2011; Perng et al., 2011b). pUL44, a HCMV DNA polymerase processivity factor that is capable of tethering the catalytic subunit pUL54 onto DNA to allow continuous elongation of the DNA strand, binds to DNA and pUL54 at two sites located within the N-terminal 309 residues of the UL44 amino acid sequence (Weiland et al., 1994; Loregian et al., 2003). Dimerization of pUL44 is critical for its function, and disruption of dimer formation blocks viral progeny production (Sinigalia et al., 2008). The nuclear localization signal (NLS) of pUL44 directs its translocation to the nucleus immediately after dimerization (Alvisi et al., 2011). Cellular protein kinase triggers phosphorylation at several lysine residues upstream of NLS in the C-terminal domain of pUL44. Phosphorylation promotes pUL44 nuclear transport, whereas another phosphorylation site, T427, inhibits nuclear targeting once phosphorylated (Alvisi et al., 2011).

An enzyme called ubc9 mediates pUL44 SUMOylation in which pUL44 is covalently conjugated to a small ubiquitin-related modifier (SUMO) molecule in the presence of DNA, and overexpression of SUMO-1 results in increased viral production (Sinigalia et al., 2012), although the effects of sumoylated pUL44 on replication remain unexamined. The peak amounts of sumoylated pUL44 were detected in the late phase (Sinigalia et al., 2012). Moreover, three transcription start sites of *UL44* were identified, with the proximal and distal promoter being activated in early infection. The middle one initiates *UL44* expression in late infection (Leach and Mocarski, 1989; Isomura et al., 2008). Additional roles of both pUL44 and sumoylated pUL44 are indicated but remain unidentified other than replication (Isomura et al., 2008; Sinigalia et al., 2012). pUL44 also shows direct interactions with SWI/SNF, a chromatin remodeling complex. The formation of replication and transcription complexes prior to DNA packaging into the capsid is facilitated by chromatin remodeling processes (Ranneberg-Nilsen et al., 2012).

*UL54* is a prototypical early gene that encodes DNA polymerase catalytic enzyme, which is undoubtedly one of the most important proteins during the viral lytic cycle. pUL54 contains different domains that confer it polymerase, 3′ to 5′ exonuclease, as well as ribonuclease H activities (Zarrouk et al., 2017). *IR1*, an 18-bp gene region, and activating transcription factor (ATF)-1, a transcription factor, contribute to the activation of *UL54* gene expression at early and late times of infection, respectively, possibly via interactions with other host and viral proteins (Kerry et al., 1994, 1997).

In addition to those six core replication proteins, IE2, UL36-38, and UL84 proteins were thought to be necessary for *Ori*Lyt-dependent DNA replication (Sarisky and Hayward, 1996). However, UL36-38 gene products were demonstrated to be unnecessary thereafter, at least in telomerase-immobilized fibroblasts (Xu et al., 2004). pUL84 interacts with IE2 (Spector and Tevethia, 1994), resulting in transdominant inhibition of IE2, decreased early gene expression, and thus reduced DNA replication (Gebert et al., 1997). Xu et al. (2004) then reported that an active promotor within *ori*Lyt was repressed by IE2, and this negative effect was rescued by pUL84. pUL84 has also been shown to directly interact with transcriptional binding sites within *ori*Lyt (Kagele et al., 2009). Moreover, pUL84 associates with pUL44 and nucleolin, and colocalizes with the proteins in the replication compartments at early times of infection (Bender et al., 2014). Posttranslational phosphorylation was also observed when pUL84 interacted with cellular casein kinase 2, and this association was required for DNA amplification (Gao and Pari, 2009). Together, pUL84 and IE2 are essential for *ori*Lyt-dependent DNA replication.

*UL45* gene encodes an inactive homolog of the large subunit of ribonucleotide reductase (RR) that catalyzes the reaction of dNTP synthesis (Patrone et al., 2003). dNTP is essential for DNA replication, but whether the deletion of *UL45* causes viral growth defects is still controversial. The virus may develop other strategies to synthesize dNTP (Hahn et al., 2002; Patrone et al., 2003).

#### Protein Expression

pUL69 is a member of the infected cell protein 27 (ICP27) family, and it contains a conserved folded domain termed ICP27 homology domain (IHD) that is required for mRNA nuclear export (Winkler et al., 1994). Although pUL69 is able to bind to RNA, RNA binding is not critical for unspliced RNA export (Toth et al., 2006). Interactions with DEXD/H-box RNA helicases UAP56 and URH49 are essential for pUL69-mediated mRNA export (Lischka et al., 2006). pUL69 can be phosphorylated by cyclin-dependent kinase (CDK)-9 and pUL97 (Graf et al., 2016). The accurate nuclear localization is attributed to the phosphorylation of pUL69 (Graf et al., 2016). Inhibition of CDK activity leads to the blockade of mRNA export (Rechter et al., 2009).

Unlike most viruses that prevent cellular protein synthesis, cellular translation proceeds in HCMV-infected cells. Therefore, HCMV usurps translational factors from the host. pUL38 recruits and accumulates translational factors by regulating poly(A) binding protein (PABA), a protein that enhances the assembly of eukaryotic translation initiation factor 4F (elF4F) (McKinney et al., 2012). pUL69 promotes translation by interacting with 4EBP1, preventing 4EBP1 binding to cap-binding complex, and thus relieving negative effects of 4EBP1 (Aoyagi et al., 2010). RNA-dependent protein kinase R (PKR) recognizes viral dsRNA, and PKR is dimerized and autophosphorylated to be activated upon engagement. The active form of PKR phosphorylates eukaryotic initiation factor 2α (eIF2α), which inhibits its function in the initiation of translation. Therefore, PKR serves as an additional defense mechanism that reduces viral synthesis. HCMV counteracts this translation repression effect by expressing pTRS1 and pIRS1 that bind to PKR followed by PKR inactivation (Ziehr et al., 2016).

#### Nuclear Egress

Nuclear egress is a process of transporting capsid containing newly synthesized and packaged viral genome out of the nucleus. HCMV wraps its genome into capsid to form the nucleocapsid, and insertion of viral genome into procapsid requires terminases (pUL56 and pUL89) that cleave two genomes in a concatemer. *UL77* encodes a capsid-associated structural protein that can bind to dsDNA and terminase subunits (Meissner et al., 2011; Köppen-Rung et al., 2016), and it has been demonstrated that pUL77 is necessary for viral DNA cleavage and packaging (DeRussy and Tandon, 2015; Borst et al., 2016). pUL76 regulates the expression of *UL77*, but whether pUL76 has effects on nucleocapsid assembly is not known (Isomura et al., 2010). It has been shown that pUL93 and portal protein pUL104 also participate in DNA package (Köppen-Rung et al., 2016).

Nuclear egress complex (NEC), a heterodimer that consists of pUL50 and pUL53, transits nucleocapsid from the nucleus to the cytoplasm (Lye et al., 2015). Restriction of *UL53* gene expression and abnormal localization of pUL50 block nuclear egress in p53KO cells (Kuan et al., 2016). NEC recruits pUL97, a protein kinase, to the nuclear rim where pUL50, pUL53, and pUL97 colocalize (Sharma et al., 2014). Lamin A/C, the components of the nuclear lamina, are phosphorylated by pUL97, which are then disrupted to allow the escape of the nucleocapsid from the inner nuclear membrane (Azzeh et al., 2006; Sharma et al., 2014, 2015). Deletion of immunoglobulin heavy chain binding protein (BiP), an ER chaperone, gives rise to abnormal lamin phosphorylation at late time, implying that BiP is also involved in nuclear egress (Buchkovich et al., 2010). The nucleocapsid budding across the inner nuclear membrane allows it to acquire a primary envelope and subsequently remove its envelope when budding through the outer nuclear membrane. Hence, it is commonly believed that two processes, envelopment and de-envelopment, are essential for nuclear egress (Mettenleiter, 2002; Goldberg et al., 2011). pUL96 assists pp150 to costabilize nucleocapsids during translocation from the nucleus to the cytoplasmic assembly compartment (Tandon and Mocarski, 2011).

#### Viral Assembly

The viral assembly complex (vAC), a juxtanuclear structure in the cytoplasm, is the site of viral tegumentation and envelopment, resulting in the formation of mature virus (Sanchez et al., 2000). The biogenesis of vAC is associated with the budding process from the secretory system, and viral pUL48 and pUL103 have been proposed to impact vAC development (Das et al., 2014). The assembly site is largely altered from a compact structure to diffuse vacuoles in the absence of functional pUL97, indicating that *UL97* plays a role in vAC organization (Azzeh et al., 2006). pUL94 is in complex with pUL97 in vAC, and the interaction may contribute to viral assembly (Liu et al., 2009).

pUL94 allows correct localization and accumulation of pp28 in vAC (Phillips et al., 2012). A threshold accumulation of pp28 is required for multimerization and viral envelopmentation (Seo and Britt, 2007, 2008). Deletion of *UL99* leads to the production of non-enveloped viral particles (Silva et al., 2003). *UL26*-deleted virions also show hypophosphorylation of UL99-encoded pp28. Abnormal phosphorylation destabilizes pp28 (Munger et al., 2006), and stability of the intact viral particle is affected (Lorz et al., 2006).

pp150–nucleocapsid complex transported to vAC is dependent on the microtubule motor system, in which BicD1 protein links pp150 (acting as a cargo) and dynein (that is a motor protein), and pp150 is actively transported in a monodirectional fashion (Indran et al., 2010). pUL71 contributes to the final step of the secondary envelopment of nucleocapsid. The oligomerization catalyzed by a basic leucine zipper-like domain on pUL71 is necessary for final envelopment (Meissner et al., 2012). The viral particle can be wrapped by pUL71, which directs the complex to transport between the *trans*-Golgi network, viral assembly compartment, budding into multivesicular bodies and plasma membrane. pUL71 is localized toward the cytosolic phase of the cell membrane and covers recycling endocytic vesicles (Schauflinger et al., 2011; Dietz et al., 2018).

UL35 protein is also localized in the perinuclear region (Liu and Biegalke, 2002). More evidence based on expression of the *UL35* mutant virus in transfected cells has revealed decreased production of enveloped particles and disappearance of dense bodies (DBs), indicating a role of pUL35 in envelopment (Schierling et al., 2005). Furthermore, the transport of envelope glycoprotein B is dependent on the binding of pUL35 and its interacting partner, sorting nexin 5 (SNX5) (Maschkowitz et al., 2018).

Three virus-related particles are formed and secreted from HCMV-infected cells. The first is the virion that contains the whole viral structure. Non-infectious enveloped particles that lack a viral genome are only produced in small amounts. DBs are commonly detected as incomplete viral particles that package envelope glycoproteins and some tegument proteins (Irmiere and Gibson, 1983). pp65 (pUL83) is abundant in DBs (Varnum et al., 2004). It was reported that DBs were able to induce apoptosis of Mo7e cells (Sindre et al., 2000). *In vivo* study revealed that DBs could elicit production of antibodies against glycoproteins such as gH and gB as well as tegument proteins pp65 and pp150 (Pepperl et al., 2000). Moreover, DB-delivered pp65 could be presented by MHC-I, and this antigen presentation promoted cellular immune responses (Pepperl et al., 2000). DBs have also been shown to facilitate the maturation of dendritic cells and their capacity for antigen uptake and presentation (Sauer et al., 2013). MiRNAs were detected within DBs and could be delivered into transfected cells (Mohammad et al., 2017). Recombinant DBs are vaccine candidates with efficient T cell and antibody responses (Becke et al., 2010).

#### Anti-apoptosis

Fibroblasts infected with HCMV lacking the *UL38* coding sequence undergo extensively morphological changes and eventual apoptosis, prompting a role for pUL38 in preventing premature cell death (Terhune et al., 2007). pUL38 binds to ubiquitin-specific protease 24 (USP24), a protein that mediates autophagic ferritin degradation in lysosomes. This association inhibits cell death as a result of the protection of lysosome integrity because the disturbance of iron homeostasis leads to lysosome instability (Sun et al., 2018). It has also been reported that pUL38 can relieve endoplasmic reticulum (ER)-mediated cell death independent of mTORC1 (Xuan et al., 2009; Qian et al., 2011). *UL36* encodes viral inhibitor of caspase-8 activation (vICA), which modulates cell death in diverse pathways. pUL36 interacts with procaspase-8 and inhibits the formation of activated caspase-8 dimer in response to Fas-mediated apoptosis (Skaletskaya et al., 2001). It is also involved in caspase-independent apoptosis with unclear mechanisms (McCormick et al., 2010). pUL44 and IE86 act concomitantly to block p53 transcriptional activity, thus overcoming cell cycle arrest and apoptosis to accomplish cellular DNA synthesis and protein production (Kwon et al., 2012).

### Envelope (Table 3)

Human cytomegalovirus is able to infect diverse cell types such as epithelial cells, fibroblasts, lymphocytes, monocytes, and macrophages, and this characterization of broad tropism is attributed to the envelope proteins that are believed to have roles in recognition, attachment, and fusion. Moreover, envelope mediators are the key to understanding cell-to-cell spread and HCMV dissemination. Entry into different cell types might be associated with concomitant effects from different combinations of envelope proteins. Three complexes termed glycoprotein complex I, II, and III (gCI, gCII, and gCIII) are present on the HCMV envelope.

**TABLE 3:** HCMV envelope proteins.

Protein	Mapped gene	Composition	Function(s)	References
gB	*UL55*	gCI	Recognition Attachment Fusion Viral entry Induction of humoral immune response	Vey et al., 1995; Smuda et al., 1997
gM	*UL100*	gCII		Li et al., 1995; Varnum et al., 2004
gN	*UL73*	gCII		Dal Monte et al., 2001; Varnum et al., 2004
gH	*UL75*	gCIII		Huber and Compton, 1999
gL	*UL115*	gCIII		Huber and Compton, 1999
gO	*UL74*	gCIII		Huber and Compton, 1999
gpUL128, gpUL130. gpUL131	*UL128, UL130, UL131*	In complex with gH/gL on envelop		Ryckman et al., 2008; Tao et al., 2014
gpUL116	*UL116*	Form complex with gH on envelop		Caló et al., 2016
gpRL13	*RL13*		Binds to IgG Fcγ	Stanton et al., 2010; Li et al., 2011; Cortese et al., 2012
gpTRL10	*RL10*		Unclear	Weekes et al., 2014; Foglierini et al., 2019
gp1	*UL1*		Has impacts on viral growth in epithelial cells but not fibroblasts	Shikhagaie et al., 2012
gp48	*UL4*		Has no impact on viral growth in fibroblasts	Hobom et al., 2000
gp42	*UL132*		Has impacts on viral growth in fibroblasts Endocytosis of gp42 into virion is required for efficient replication	Spaderna et al., 2005; Kropff et al., 2010

#### gCI

gCI comprises two subunits, gp58 and gp116, which are the products of furin protease-cleaved glycoprotein B (gB), a precursor of 160 kDa (Vey et al., 1995). gB has been shown to play roles in viral entry into permissive cells (Isaacson and Compton, 2009) and as a target of neutralizing antibodies (Tabata et al., 2019). It contains a disintegrin-like domain that recognizes integrins, and the integrin signals are likely to promote fusion since the delivery of pp65 is increased in the presence of gB disintegrin-like peptide, but attachment remains unaffected (Feire et al., 2004). Binding of gB also triggers alteration of host signaling pathways. Upon binding, induced expression of myeloid cell leukemia (Mcl)-1 protein enhances antiapoptotic effect in non-permissive progenitor myeloid cells (Reeves et al., 2012). Small interfering RNA targeting Mcl1 exhibit increased cell death of HCMV-infected monocytes in comparison to mock infection and use of control siRNA (Chan et al., 2010). Moreover, blockade of epithelial growth factor receptor (EGFR) with specific neutralizing antibodies and pharmacological inhibitors results in the downregulation of Mcl-1 gene expression and shows similar level of cell viability to mock infection. Therefore, EGFR in monocytes appears to be an important receptor that interacts with HCMV. EGFR is further proved to be required for mediating viral entry into CD34^+^ progenitor cells (Kim et al., 2017). In fibroblasts, however, HCMV infection fails to stimulate EGFR phosphorylation and activation (Isaacson et al., 2007). Viral entry is not affected in fibroblasts, epithelial and endothelial cells pretreated with EGFR antibodies (Isaacson et al., 2007). The roles of EGFR are still controversial. Further, host cells, at least monocytes, upregulate inflammatory gene expression such as interleukin-1β (IL-1β) upon gB engagement (Yurochko and Huang, 1999).

There are various polymorphic sites along the gB-encoded *UL55* gene containing 906 amino acids, with the most variable regions present in the N-terminus, C-terminus, and furin cleavage sites (Stangherlin et al., 2017). It has been demonstrated that the genotype of gB differs with respect to the geographic distribution (Zipeto et al., 1998). For example, compared with patients from California, an increase in the gB4 genotype has been observed in immunocompromised patients from Italy (Zipeto et al., 1998). Infection by HCMV with different gB genotypes may not share a comparable natural disease course (Roubalová et al., 2010). gB1 and gB2 types are often seen in patients with transplantation and infection with human immunodeficiency virus (HIV), respectively (Fries et al., 1994; Shepp et al., 1996). The 275Y variants of gB expressed on the AD169 strain promote fusogenicity with activation of caspase 2 and the DNA damage response since the 275D variants show less syncytium formation and an inability to trigger caspase 2 (Tang et al., 2019). Whether gB genotypes are associated with cell tropism are largely elusive.

#### gCII

gCII is designated as a complex that is formed covalently and non-covalently by gM together with gN, which are the products of *UL100* and *UL73* ORFs, respectively (Mach et al., 2000; Varnum et al., 2004). Formation of the gM/gN complex in the ER is required for transport and intracellular trafficking of the heterodimer to the mature virion assembly center (Mach et al., 2005). Binding to cell surface heparan sulfate proteoglycans (HSPGs) is the initial step in which virus tethers and attaches to the host (Compton et al., 1993). gCII (Kari and Gehrz, 1992) and soluble gB (Boyle and Compton, 1998) are interaction partners of heparin, and the interactions thus facilitate viral attachment and entry. Similar to gB, gM and gN elicit humoral responses in which neutralizing antibodies are produced (Shen et al., 2007). Anti-gM/gN antibodies may be able to recognize HCMV unlimited by strain specification since anti-gM/gN IgG antibodies generated from AD169 can bind to the Toledo strain (Shimamura et al., 2006).

#### gCIII

gCIII contains the gH/gL/gO complex, a high-molecular-weight complex (Kinzler et al., 2002). This is further complicated by the fact that co-expression of the *UL75* (gH), *UL115* (gL), and *UL74* (gO) genes does not readily result in the formation of the tripartite complex (Huber and Compton, 1999). gH binding triggers activation of transcription factors such as NK-κB, a process that can be blocked by anti-gH neutralizing antibodies (Yurochko et al., 1997). The *UL74* sequence shows variations, leading to a growing recognition of the functional importance of gO (Paterson et al., 2002). Further studies have described an increased sensitivity of *UL74*-deleted HCMV-infected fibroblasts in the context of anti-HCMV serum, anti-gB antibodies, and anti-gH antibodies (Jiang et al., 2011). In addition to the gH/gL/gO complex, gH/gL interact with UL128–131 gene products. The different constitutions of the two complexes are associated with cell-specific tropism. The gH/gL/gO complex is required for entry and dissemination between fibroblasts, while viral entry into epithelial and endothelial cells requires the gH/gL/UL128–131 complex to bind to cognate receptors (Huber and Compton, 1998; Wang and Shenk, 2005; Adler et al., 2006). In addition, UL128–131A gene products are required for efficient internalization into monocytes via integrin-mediated signaling (Nogalski et al., 2013), and they are crucial for infection of monocytes (Straschewski et al., 2011) and dendritic cells (Gerna et al., 2005).

#### Other Proteins

Stanton et al. (2010) assumed that the highly glycosylated RL13 protein may be a surface envelope protein. Its role on envelope was further studied by another group (Cortese et al., 2012). Fcγ signals could be detected though flow cytometry in *RL13*-expressed HEK293T cells that were exposed to DyLight 649-conjugated human Fcγ, suggesting that RL13 interacted with Fcγ. This result was further confirmed by confocal microscopy analysis that showed colocalization of Fcγ and RL13 (Cortese et al., 2012). Therefore, RL13 has Fc binding abilities. Four viral encoded G-protein coupled receptors (GPCRs) are also viral membrane proteins, which have been intensively reviewed (Krishna et al., 2018; Frank et al., 2019). Their functions are briefly summarized in Table 4.

**TABLE 4:** Viral GPCRs.

Protein	Mapped gene	Function(s)	References
pUL33	*UL33*	➢ Co-localizes, interacts and further blocks US28-induced NK-κB activation ➢ Enhances transcription mediated by cAMP responsive element ➢ Forms a complex with pUL78, host CCR5 and CXCR4, and impairs CXCR4 effects ➢ Has oncomodulatory properties	Fraile-Ramos et al., 2002; Casarosa et al., 2003; Tschische et al., 2011; Tadagaki et al., 2012; van Senten et al., 2019
pUL78	*UL78*	➢ Co-localizes, interacts and further blocks US28-induced NK-κB activation ➢ Form a complex with pUL33, host CCR5 and CXCR4, and impairs CXCR4 effects ➢ Has impacts on viral growth in epithelial and endothelial cells but not fibroblasts	Michel et al., 2005; Tschische et al., 2011; O’Connor and Shenk, 2012
gpUS27	*US27*	➢ Promotes CXCL12/CXCR4 signaling ➢ Increases surface CXCR4 level ➢ Facilitates CXCR4 internalization after CXCL12 binds ➢ Stimulates expression of stress response genes ➢ Increases DNA replication ➢ Induces pro-survival factors such as Bcl-x	Fraile-Ramos et al., 2002; Lares et al., 2013; Boeck and Spencer, 2017; Boeck et al., 2018; Tu et al., 2018
pUS28	*US28*	➢ Acts as chemokine receptors, has multiple ligands and activates multiple signaling pathways ➢ Chemokine internalization ➢ Promotes survival ➢ Activates MIEP in differentiated cells ➢ Promotes latency in undifferentiated cells	Fraile-Ramos et al., 2002; Krishna et al., 2017a, 2018; Zhu et al., 2018; Frank et al., 2019

## Different Significances of Virion-Carried Molecules in Primary Infection and Reactivation

### Roles of pp71 in Primary Infection and Reactivation (Figure 1)

Lifelong HCMV infection is attributed to its latency in healthy individuals. Single-cell transcriptomic analysis found that the transcription program in the latently infected cells mirrors that at the late phase of lytic replication, but the latency-associated expression level is low (Shnayder et al., 2018). Viral abilities to re-express lytic genes from the quiescent form in response to certain stimuli are associated with complications such as severe pneumonia and gastrointestinal disease following transplantation (Zhao et al., 2017). CD34^+^ HPCs and CD14^+^ monocytes do not provide a platform for viral replication, and undifferentiated cells are considered to be the latent reservoirs of HCMV (Mendelson et al., 1996; Khaiboullina et al., 2004). Nuclear pp71 can be detected by immunofluorescence in normal human dermal fibroblasts (NHDFs), the permissive cells for HCMV lytic replication. By contrast, pp71 is retained in the cytoplasm instead of the nucleus of HPCs (Saffert and Kalejta, 2007). Interestingly, the process of viral entry into CD34^+^ HPCs by macropinocytosis does not allow pp71 to escape from endosomes, and endosome retention is at least one possible mechanism that ensures the cytoplasmic localization of pp71 in HPCs (Lee and Kalejta, 2019). Cytoplasmic localization of pp71 has also been observed in NTERA-2 (NT2) cells that are incompletely differentiated cell lines (Penkert and Kalejta, 2010). The pp71 subcellular localization is crucial for its transactivities and therefore associated with the replicative or latent phase. The underlying mechanism is largely determined by the interaction of pp71 with components of the ND10 (Hofmann et al., 2002).

**FIGURE 1:**
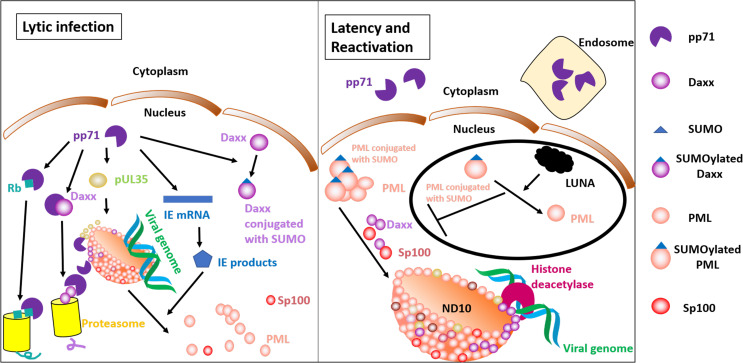
Roles of pp71 in the lytic and latent phase. The **left panel** shows lytic gene expression. Nuclear pp71 is capable of promoting pUL35 to recruit PML, an ND10 component that has impacts on correct localization of Daxx and Sp100. pp71 interacts with Daxx and induces Daxx for degradation in a proteasome-dependent manner. pp71 transactivates IE gene expression, and IE products disrupt ND10. pp71 also has abilities to induce Rb degradation, a process that contributes to relieve Rb-mediated cell cycle block. The **right panel** shows the latent state and reactivation. pp71 is retained in the cytoplasm or endosome rather than the nucleus. ND10 components represent an intrinsic defense mechanism. Daxx, PML, and bound transcription factors recruit histone deacetylases, resulting in viral gene silencing. LUNA-mediated reactivation is shown in a black circle. LUNA contains isopeptidase activity that deSUMOylates PML and disrupts PML-induced recruitment of Daxx and Sp100. Therefore, LUNA promotes reactivation.

Promyelocytic leukemia protein (PML)-associated nuclear bodies (also known as ND10), subnuclear spherical and punctate structures that are, on average, 0.3 to 0.5 μm in size (Ahn et al., 1999), are thought to be the location for input viral DNA accumulation (Ishov and Maul, 1996). Death domain-associated protein (Daxx), one of the most important components in ND10, acts as an intrinsic defense mechanism against HCMV by restriction of viral gene expression as demonstrated by the inhibition of viral replication in cells overexpressing Daxx (Cantrell and Bresnahan, 2006). Daxx-induced HCMV transcriptional repression promotes the establishment of latent states (Saffert and Kalejta, 2007). Daxx and other ND10 proteins that bind to transcriptional factors are able to recruit histone deacetylases (HDACs) (Hollenbach et al., 2002; Maul, 2008). The major immediate early promotor (MIEP)-bound histones are unacetylated in undifferentiated cells (Reeves et al., 2005), and thus MIEP that drives lytic replication is transcriptionally inert. Downregulation of Daxx results in failed formation of heterochromatin and increased IE gene expression (Woodhall et al., 2006). Moreover, Daxx together with ATRX forms a complex that is considered to be a H3.3 histone chaperone that affects H3.3 deposition on heterochromatin (Lewis et al., 2010). In addition to Daxx, other components of ND10, including PML and Sp100, also contribute to viral genome repression (Glass and Everett, 2013). Compared with the knockdown of Daxx alone using siRNA, triple deletion of Daxx, PML, and Sp100 can more efficiently inhibit gene expression and viral production (Glass and Everett, 2013). In the absence of PML, Daxx and Sp100 show a dispersed distribution and disrupted colocalization (Tavalai et al., 2006).

By two-yeast hybrid system screening (Hofmann et al., 2002) and immunoprecipitation (Hofmann et al., 2002; Ishov et al., 2002), pp71 has been found to be an interactor with Daxx. Hofmann et al. also confirmed colocalization of Daxx and pp71 (Hofmann et al., 2002). A mutant version of pp71 that lacks binding ability to Daxx is unable to transactivate IE expression (Hofmann et al., 2002). Therefore, the association of pp71 and Daxx is pivotal for the functions of pp71. pp71 proteins are accumulated in the nucleus prior to IE2 transcription (Ishov et al., 2002). The following study reported that pp71 was able to induce Daxx degradation through proteasome, independent of ubiquitin (Hwang and Kalejta, 2007). Daxx degradation is critical for pp71 functions in transactivation since Daxx plays roles in the recruitment of HDACs, heterochromatin formation, and transcriptional repression (Hollenbach et al., 2002; Woodhall et al., 2006). Acetylation of MIEP-associated histones was detected when latently infected CD34^+^ progenitor cell terminally differentiated into dendritic cell, a permissive cell type that supports the HCMV productive phase (Reeves et al., 2005). Furthermore, pp71 also promotes Daxx SUMOylation, but this posttranslational modification is unable to affect pp71 transactivity (Hwang and Kalejta, 2009). SUMOylation of PML is required to recruit Daxx (Ishov et al., 1999), but the effect of SUMOylation still needs to be investigated. The interplay between ND10 and pp71 is further complicated by evidence showing that pp71 facilitates the UL35 gene products pUL35 to form UL35 nuclear bodies, which are capable of recruiting PML, Daxx, and Sp100 (Salsman et al., 2011). The transfected cells infected with mutant virus that harbors deletions in the *UL35* gene show decreased IE gene expression (Schierling et al., 2005). UL35a protein, a short-sized UL35 gene product, however, shows reduced formation of UL35 nuclear bodies and contributes to pp71 cytoplasmic localization (Salsman et al., 2011). Indeed, previous research has reported that pUL35a inhibits pp71-mediated MIEP activation (Liu and Biegalke, 2002). However, unlike pUL35 that was found in virions, pUL35a is not packaged into the mature virion, and its transcripts are detected 4 h after infection (Liu and Biegalke, 2002). It is likely that pUL35 plays roles earlier than pUL35a as pUL35 is a preexisting protein. After pp71 degrades Daxx and activates IE gene expression, IE gene products disperse and disrupt ND10 (Korioth et al., 1996; Ishov et al., 1997). A recent study has reported that LUNA-mediated deSUMOylation is required for PML dispersal (Poole et al., 2018).

Less efficient reactivation has been detected in dendritic cells that are differentiated from CD34^+^ progenitor cells infected with LUNA-defective HCMV, compared to wild-type HCMV infection (Poole et al., 2018). In contrast, viral production in fibroblasts shows no differences between wild-type and LUNA knockdown group (Poole et al., 2018). Further, in comparison to wild type, PML knockdown results in increased numbers of cells that prime IE gene expression of fibroblasts (Tavalai et al., 2006), but no distinction in IE-forming units in the reactivation model (Poole et al., 2018). Heterogeneous cell fusion experiments in which NHDFs form syncytia with viral-infected NT2 cells showed that pp71 proteins that were previously localized in the NT2 cytoplasm were detected in nuclei in syncytia, indicating that terminally differentiated cells provide factors that promote pp71 nuclear accumulation (Penkert and Kalejta, 2010). As discussed above, pp71 cytoplasmic or nuclear localization contributes to the establishment of the latent or replicative phase, respectively. However, reactivation can occur at various times after the initial infection. This process is unlikely to utilize the pp71 retained in the cytoplasm. Moreover, latency-associated pp71 expression is not significantly high (Cheng et al., 2017). Whether pp71 is crucial for IE gene expression during reactivation is unclear, and this knowledge may hold the key to understanding HCMV-associated disease and even mortality posttransplantation. Together, there might be differences in the initiation of IE transcription in primary infection and reactivation.

### Roles of pUS28 in Primary Infection and Reactivation

pUS28, a virally expressed GPCR, shares homology with human chemokine receptor (Gao and Murphy, 1994) and is able to interact with diverse CC and CX(3)C chemokines (Gao and Murphy, 1994; Kledal et al., 1998). The *US28* gene is transcribed in both the lytic and latent phases (Beisser et al., 2001). Recent transcriptome analysis has confirmed latency-associated transcription in both natural CD34^+^ HPCs and experimentally latent models (Cheng et al., 2017). Deletion of *US28* results in the failed establishment of latency. Also, in undifferentiated monocytes, MIEP is associated with phosphorylated H3 (a histone marker of transcriptional activation) and heterochromatin protein 1 in the absence and presence of US28 respectively (Krishna et al., 2017a). These findings suggest that pUS28 plays a role in the promotion of the latent phase. Moreover, the authors further found that functional pUS28 was able to increase MIEP activities of differentiated cells (Krishna et al., 2017a). Together, these data show that pUS28 has different effects on the lytic and latent phases, and activities of pUS28 are heavily dependent on the differentiation states. The point mutation R129A in US28 results in an inability to activate or repress signaling pathways, and Y16F leads to pUS28 that is incapable of binding to chemokines. Expression of US28 with Y16F by lentivirus complements pUS28-mediated latency, whereas R129A does not, demonstrating that Y16F does not affect pUS28-induced latency, and signaling pathway alteration is linked to the effects of pUS28 on latency establishment (Krishna et al., 2017a). Further, pUS28 is likely to dephosphorylate MAPK and NK-κB factors in undifferentiated cells, at least partially contributing to the establishment of latency. It has opposite impacts on differentiated cells, namely, hyperphosphorylation of signaling molecules to facilitate lytic infection (Krishna et al., 2017a). A recent report discovered that HCMV forces HPCs into a monocyte subset characterized by a longer lifespan and immunosuppressive phenotype via STAT3-iNOS-NO cascade to achieve latency (Zhu et al., 2018). Deletion of *US28* leads to STAT3 inactivation and subsequent failure of latent establishment, indicating that pUS28-mediated STAT3 phosphorylation is essential for latency (Zhu et al., 2018).

### Roles of Virion-Carried Molecules in Nuclear Targeting During Primary Infection

Nuclear targeting is a process of transportation of incoming nucleocapsid to host nucleus, and this process is dispensable for reactivation. The bipartite NLS of pUL48 mediates nuclear targeting, a process that targets the nucleocapsid toward the host nucleus and releases the viral genome into the cell nucleus (Brock et al., 2013). pUL48 may cooperate with pUL47 to promote disassembly of the nucleocapsid and enhance the release of viral DNA from the capsid (Bechtel and Shenk, 2002). pUL47 is a pUL48-binding protein, and the interaction facilitates cleavage of isopeptide bonds via the deubiquitylating activity of pUL48 (Tullman et al., 2014). A mutant virus that lacks deubiquitinating activity shows decreased replication and dissemination as a result of reduced autodeubiquitination (Kim Y. E. et al., 2016).

Tegument proteins preserve the integrity of the capsid that houses an incompatible large genome. The 150-kDa phosphoprotein encoded by the *UL32* gene, a second abundant component of the tegumental layer, was found to interact with the triplex on the capsid and to extend toward the SCP (Yu et al., 2017). The subcellular localization of pp150—nucleus or cytoplasm—is still controversial, and it may accompany the capsid and transit from nucleus to cytoplasm (Sanchez et al., 2000; Sampaio et al., 2005). Nevertheless, pp150 is thought to be involved in nuclear targeting during primary infection, organization of the assembly compartment, tegumentation, and virion egress during late infection due to its extensive structural associations with the nucleocapsid (AuCoin et al., 2006; Tandon and Mocarski, 2008; Indran et al., 2010).

### Roles of Virion-Carried Molecules in Host Cell Cycle Regulation

Viral replication is a complex cascade that requires the host cells to provide replicative factors and nutrients, and those substances supplied during cell division are not sufficient for viral growth, especially for HCMV with a long replication cycle. Hence, HCMV develops strategies to arrest host in the G1/S phase to accomplish the whole lytic replication cycle. In primary infection, virally delivered factors can be readily used to regulate the host cell cycle.

Viral pUL21a destabilizes cyclin A2 via a conserved cyclin A2 binding motif, arginine-x-leucine (RxL), via proteasome-dependent degradation to arrest host cell cycle progression (Caffarelli et al., 2013). Overexpression of cyclin A2 renders host cells entry into mitotic phase (Eifler et al., 2014). pUL21a further modulates the host cell cycle by degrading anaphase-promoting complex/cyclosome (APC/C) subunits that serve as an E3 ubiquitin ligase that digest certain proteins in the G1 and M phase to regulate the cell cycle (Fehr et al., 2012). pp150 mutant virus that lacks binding ability to cyclin A2 is unable to arrest hosts in the S/G2 block, but cell gene expression is restricted at the G2/M phase (Weisbach et al., 2017). Double mutation of pp150 and pUL21a, both of which are unable to interact with cyclin A2, results in unrestricted G2 and mitotic entry (Weisbach et al., 2017). Those data indicate that pp150 and pUL21a concomitantly reprogram the cell cycle (Weisbach et al., 2017). In response to stress signaling such as viral infection, tuberous sclerosis protein complex 2 (TSC2) is activated, which in turn blocks mTORC1 activities, halting cell growth (Moorman et al., 2008). mTORC1 can also be activated by pUL38 in a TSC2-independent pathway, suggesting that pUL38 is able to modulate cell growth (Moorman et al., 2008; Bai et al., 2015). pUL69 has also been reported to induce G1 blockade (Lu and Shenk, 1999; Hayashi et al., 2000).

If the host is default to pass through the S/G2 phase and undergo subsequent division, HCMV replication is decreased to wait for a time that allows sufficient viral growth. Cell-cycle-associated IE gene repression is independent of intrinsic defense mechanisms such as PML (Zydek et al., 2011). The viral reproductive cycle can be inhibited with high levels of cyclin A in the S/G2 phases, and the interaction between pp150 and cyclin A is essential for inhibition (Weisbach et al., 2017). pp150–cyclin A2–CDK activities in the S/G2 phases interfere with viral IE gene expression by blocking IE gene expression and affecting IE mRNA splicing (Oduro et al., 2012; Bogdanow et al., 2013). Therefore, once the sensor protein pp150 binds to cyclin A, HCMV transiently shuts down IE gene expression to ensure that viral lytic replication does not take place in the time course of host cell division (Bogdanow et al., 2013).

In the quiescent state, retinoblastoma (Rb) forms a complex with E2F, inhibiting the expression of E2F-responsive genes and subsequent cell cycle progression. Rb (Hume et al., 2008) and its family members (Iwahori et al., 2017) are phosphorylated by viral pUL97. Hyperphosphorylation and inactivation of Rb by pUL97 activates E2F-mediated transcription and cell cycle progression (Iwahori et al., 2015). Moreover, Rb interacts with ND10-associated proteins (Alcalay et al., 1998), enhancing the transcriptional repression of the viral genome (Fang et al., 2002). Hyperphosphorylation of Rb by pUL97 relieves this negative effect in a PML-dependent manner (Fang et al., 2002). Additional mechanisms are also involved in the regulation of Rb family members. pp71 degrades Rb proteins by the proteasome (Figure 1; Kalejta and Shenk, 2003).

Reactivation is highly dependent on cell differentiation states. Upon treatment of interleukin-4 (IL-4) and granulocyte macrophage/colony stimulating factor (GM/CSF), experimentally latent CD14^+^ monocytes differentiate into immature dendritic cells (DCs) that do not display robust lytic replication (Reeves and Compton, 2011). Additional treatment of lipopolysaccharide (LPS) and interleukin-6 (IL-6) transforms immature cells into mature DCs that support HCMV reactivation, suggesting that differentiation and inflammation are two leading stimuli for viral reactivation (Reeves and Compton, 2011). Thus, reactivation occurs in final differentiated cells and relatively independent of regulation of the host cell cycle.

## HCMV Protein Related Anti-Viral Therapy

The most common antiviral drugs used to control HCMV infection are ganciclovir (GCV), valganciclovir, foscarnet, and cidofovir. Valganciclovir, the prodrug of ganciclovir, can be metabolized to GCV, an analog of nucleoside guanosine. GCV is activated by phosphorylation catalyzed by pUL97 and cellular kinases to form ganciclovir triphosphate, terminating viral DNA synthesis by incorporation into the newly produced DNA strand and inhibition of pUL54 DNA polymerase activities (Matthews and Boehme, 1988). Therefore, mutations in *UL54* or *UL97* or both give rise to drug resistance, with more cases related to *UL97* mutations, for example, deletions of codon 594 in the UL97 genes (Keyvani et al., 2016) and point mutations such as M460V/I, H520Q, N510S, C592G and C603W (Cocohoba and McNicholl, 2002; Bachmann et al., 2013). Cidofovir is an analog of deoxycytidine monophosphate, which is further phosphorylated to a deoxycytidine triphosphate analog. Post-hematopoietic stem cell transplantation (HSCT) patients generally do not benefit from brincidofovir, a lipid conjugated cidofovir, applied for HCMV prophylaxis. All-cause mortality was 15.5 and 10.1% in the brincidofovir and placebo group, respectively (Marty et al., 2019). A similar approach is employed by cidofovir triphosphate to block pUL54 activities. Foscarnet is also an alternative choice because it inhibits pyrophosphate binding site on pUL54, and thus, pyrophosphate is not able to be cleaved from nucleotides. These available antiviral agents are limited by resistance, poor bioavailability as well as acute and long-term adverse effects such as severe myelosuppression. Most anti-HCMV drugs in widespread use were developed to target pUL54 polymerase, while maribavir interacts with pUL97 and disrupts the kinase activities of pUL97 (Razonable, 2018). Maribavir had comparable effects to valganciclovir in the control of HCMV viremia in the posttransplantation settings in a phase two trial (Maertens et al., 2019). However, it is difficult to determine or distinguish whether the HCMV viremia results from primary infection or reactivation. Therefore, whether the drug has different efficacies in primary infection and reactivation are unknown. Letermovir targets pUL56, the large subunit of the terminase complex that plays roles in DNA cleavage/packaging (Ligat et al., 2018). In a phase 2 clinical trial that administered letermovir as prophylaxis therapy to HCMV-seropositive patients after HSCT, letermovir (at a dose of 120 and 240 mg per day) was shown to significantly reduce HCMV reactivation compared with placebo (Chemaly et al., 2014). ASP0113, a DNA-based vaccine, contains plasmids encoding gB and pp65 (Smith et al., 2013). However, the prevention efficacy is not significant in D^+^ /R− kidney transplantation (Vincenti et al., 2018). gB vaccine with MF59 adjuvant has been reported to reduce congenital primary infection (Pass et al., 2009). Moreover, gB vaccine was also effective for decreasing reactivation rates in D^+^ /R− post HSCT (Griffiths et al., 2011).

New methods have been developed following advances in molecular biology. *In vitro* studies have shown a promising therapeutic method using siRNAs targeting gene transcripts of *UL54*, *UL97*, and *UL122/123*. Despite the high efficacy *in vitro*, no *in vivo* analysis has been published (Hamilton et al., 2014). However, other studies have demonstrated that siRNA targeting *UL54* is less effective and has limited utility (Shin et al., 2006). Viral GPCR pUS28 is expressed at the host membrane to interact with chemokines and triggers internalization once engaged. F49A-fusion toxin proteins (F49A-FTP) can bind to pUS28 to subsequently mediate endocytosis. The coupled toxin has cytotoxic effects on target cells. This novel strategy was shown to efficiently kill targets with lytic infection (Spiess et al., 2015) and to reduce reactivation of cells latently infected with HCMV (Krishna et al., 2017b).

## Summary

Human cytomegalovirus infects large populations and becomes dormant in primitive progenitor cells. Reactivation in transplant recipients can lead to severe CMV disease and cause death. Usage of virion-carried mediators (especially surface glycoproteins and tegument proteins) is associated with biological processes during primary infection, latency, and reactivation. Viral envelope proteins determine cell tropism in primary infection and dissemination. They act as targets for neutralizing antibodies and vaccines. Tegument links the envelope and nucleocapsid. Although the limited amounts of some tegument proteins are not sufficient to complete associated biological processes, they still participate in viral life cycle and merit discussion. Unlike primary infection in which virion-carried proteins can assist HCMV to reconcile the host to favor viral proliferation, they cannot be readily used by viruses in the context of reactivation. Those packaged proteins such as pp71 are differentially used in primary infection and reactivation. Some biological processes, such as nuclear targeting, are unique to primary infection, and thus proteins involved in such processes predominantly play roles in primary infection rather than reactivation. The mechanism by which HCMV rapidly produces molecules that enhance re-expression of lytic genes holds the key to understanding reactivation. Moreover, although the virus becomes latent in HPCs upon initial infection, the strategies used by HCMV to dampen immune response toward virion-delivered foreign molecules such as glycoproteins on envelope and tegument proteins remain to be understood.

## Author Contributions

Y-QW wrote the manuscript. X-YZ outlined the manuscript and made a deep intellectual contribution to the work. Both authors listed approved the final version of the manuscript.

## Conflict of Interest

The authors declare that the research was conducted in the absence of any commercial or financial relationships that could be construed as a potential conflict of interest.
